# Implementation and Cost Analysis of a Novel Silicosis Case-Finding Program For Mine Workers in Rural Rwanda

**DOI:** 10.9745/GHSP-D-23-00290

**Published:** 2024-04-29

**Authors:** Robert Tumusime, Michael S. Miller, Anne Niyigena, Symaque Dusabeyezu, Pierrot Uwitonze, Emmanuel Harerimana, Grace Umugiraneza, Wellars Dusingizimana, Samuel Hatfield, Stella Savarimuthu, Juliana Lawrence, Pacifique Hagenimana, Jean Marie Vianney Ngenzi, Aristarque Murara, Phoebe Mwiseneza, Paul Sonenthal, Vincent K. Cubaka, Fredrick Kateera, Innocent Kamali

**Affiliations:** a Partners In Health-Rwanda/Inshuti Mu Buzima, Rwinkwavu, Rwanda.; b Department of Medicine, Brigham and Women’s Hospital, Boston, MA, USA.; c Ministry of Health of Rwanda, Kigali, Rwanda.; d Rwinkwavu District Hospital, Kayonza, Rwanda.; e Department of Medicine, University of California at San Francisco, San Francisco, CA, USA.; f Department of Medicine, Yale New Haven Hospital, New Haven, CT, USA.; g Wolfram Mining and Processing Ltd, Kigali, Rwanda.; h Division of Pulmonary and Critical Care Medicine, Brigham and Women’s Hospital, Boston, MA, USA.; i Harvard Medical School, Boston, MA, USA.; j Partners In Health, Boston, MA, USA.

## Abstract

Little is known about the burden of silicosis in Africa, despite extensive mining and construction operations in the region putting numerous people at risk. The implementation experience and costs of case-finding for occupational lung disease in resource-limited settings are also currently unknown. We describe the first-ever silicosis case-finding project in rural Rwanda using chest X-ray, symptom questionnaires, and spirometry. This was coupled with routine noncommunicable disease case-finding for diabetes and hypertension. We performed an ingredient-based analysis of the costs of all case-finding activities. In 2022, over 25 days, 1,032 mine workers were included in the program, of which 1,014 (98.3%) completed silicosis case-finding activities. The total cost of the program was estimated to be US$38,656, representing a cost of US$37.49 per person. We conclude that conducting large-scale occupational lung disease case-finding is clinically and economically feasible in resource-limited settings and can be effectively integrated with routine noncommunicable disease case-finding.

## Abstract (precis)

Implementing occupational lung disease case-finding in resource-limited settings is clinically and economically feasible and can be integrated into routine noncommunicable disease case-finding.

## Teaser

**Key Findings:**

- A novel case-finding program for silicosis was developed and implemented for mine workers working in semi-industrial mines that included education about causes and protection, symptom screening for silicosis and TB, chest X-ray, spirometry, and screening for hypertension and diabetes.
- Of the 1,032 mine workers enrolled in the program, 95 (9.2%) had radiographic evidence of silicosis, 221 (21.6%) screened positive for hypertension, and 12 (1.1%) had elevated glucose readings.
- Partnership between private employers, the Ministry of Health, and nongovernmental organizations is essential for occupational lung disease case-finding and linkage to care.

**Key Implication:**

- Occupational lung disease case-finding can be integrated with routine noncommunicable disease case-finding to reduce costs and improve comprehensive linkage to care.

## INTRODUCTION

Silicosis is an incurable interstitial lung disease caused by the inhalation of respirable crystalline silica (i.e., silicon dioxide) particles.^1^ Silicon is among the most abundant elements in the earth’s crust, and silicosis has been observed in workers from a wide range of industries, including but not limited to construction, stone-cutting, farming, textiles, and dental prostheses.^2^ Mining has a well-established association with silicosis. Silica is present in nearly all mining operations, and the highest rates of silicosis-related deaths often occur in areas with mining activities.

Despite efforts from United Nations agencies like the World Health Organization and the International Labor Organization (ILO) to eliminate silicosis by 2030, it remains a global problem.^3^^–^^5^ The 2017 Global Burden of Disease study estimated an annualized incidence rate of 23,695 cases, which, based on the 2019 study, corresponded to an estimated 12,900 deaths annually.^2^^,^^6^ Both numbers are felt to underestimate the true disease burden, particularly in low- and middle-income countries (LMICs), where few governments have legislated exposure limits, and little has been done to quantify the degree of exposure.^7^^,^^8^ While the global age-standardized incidence rate of silicosis has decreased by an annual average of 0.8% over the last 3 decades, these improvements have not been distributed evenly. Countries with a high sociodemographic index experienced a 2.2% average annual decrease compared to 0.5% in countries with a low sociodemographic index, reflecting a significant divergence in the global response to silicosis, at least in part due to differences in regulatory standards.^2^

Little is known about the burden of silicosis in sub-Saharan Africa despite extensive mining operations in the region. Disease surveillance has been limited to southern Africa, where estimates vary considerably. In South Africa, the prevalence has been estimated to be approximately 5% among gold miners based on survey and chest X-ray screening, which underestimates the 15% prevalence detected on autopsy surveillance.^9^^,^^10^ Studies from Lesotho and Botswana using questionnaires, spirometry, and chest X-rays estimated the prevalence of pneumoconiosis to be 24% and 27%, respectively.^11^^,^^12^ To our knowledge, few studies to date have attempted to estimate the prevalence of silicosis in East Africa despite reports of occupational exposures reaching 300-fold greater than the recommended limit.^13^^–^^15^ Furthermore, despite the demonstrable need, the process and costs of implementing occupational lung disease screening in low-resource settings are not well established in the literature.

Little is known about the burden of silicosis in sub-Saharan Africa despite extensive mining operations in the region.

In this article, we describe the implementation of the first-ever case-finding program to detect silicosis among small-scale and semi-industrial mine workers in rural Rwanda, including the training of staff in spirometry, the case-finding process, the cost of the program, and our ongoing efforts to link participants to clinical care. In addition to identifying silicosis, we also detail our integration of routine noncommunicable disease (NCD) case-finding for diabetes and hypertension into the program to reduce total costs and provide more comprehensive linkage to care. By sharing our programmatic experiences, we hope to demonstrate both the feasibility and importance of case-finding programs for silicosis among workers in high-risk professions.

## SILICOSIS CASE-FINDING PROGRAM DESCRIPTION

### Setting and Stakeholders

Since 2008, Wolfram Mining and Processing Ltd. (WMP) has overseen mining operations in Rwinkwavu, Kayonza district.^16^ Kayonza is in the eastern province of Rwanda, bordering Tanzania, with a population of approximately 350,000 people.^17^ WMP operates artisanal and semi-industrial mines and conducts both surface and underground mining operations, extracting primarily cassiterite, which is the world’s largest source of tin.^16^ They operate 8 mining sites in Kayonza district and are among the largest employers in the district with approximately 1,100 employees, mainly through subcontracting with miners. Miners perform functions such as in-hole drilling, rock-blasting, manual excavation of rock, and mineral sorting and washing.

Most of the mine workers at WMP receive health care within the catchment area of Rwinkwavu District Hospital (RDH), which is located in southern Kayonza district. It has a 40-bed internal medicine ward serving a catchment population of more than 350,000 people. There is 1 full-time internist supervising numerous general practitioners. The hospital is run by the Ministry of Health (MOH) of Rwanda, and it receives support from Partners In Health/Inshuti Mu Buzima (PIH/IMB), a nongovernmental organization dedicated to increasing access to health care and improving health system strengthening in Rwanda since 2005. PIH/IMB partners with the MOH of Rwanda to support health system strengthening in 3 rural district hospitals, including RDH.

In 2021, WMP approached PIH/IMB requesting assistance with a silicosis case-finding program among their mine workers, to which PIH/IMB responded positively in 2022, as the request fell squarely under its mandate. PIH/IMB then received approval from the Director General of RDH to proceed with the partnership. All mine workers from the 8 mining sites operated by WMP in Kayonza district were invited to participate in the case-finding program.

### Staff and Training

A total staff of 25 staff members contributed to the design and implementation of the screening program. Paid PIH/IMB and MOH staff included 7 nurses, 2 data collectors, 1 study coordinator, 1 research manager, 1 NCD program manager, 1 NCD director, 1 Rwandan-based radiologist, 2 radiology technicians, and 1 Rwandan-based internist physician working at RDH. A volunteer staff, composed of 2 U.S.-based radiologists, a pulmonologist, and 3 internists, remotely assisted with program design, training, and interpretation of spirometry and chest X-ray results.

Data collectors and nurses were trained to use both a paper-based questionnaire and the Research Electronic Data Capture (REDCap) mobile application, which was loaded on tablets during screening activities.^18^ A spirometry training curriculum for all nurses was developed and implemented by a pulmonologist-led team of physicians. This consisted of 2 full-day training sessions on the theory, indications, contraindications, and interpretation of spirometry, led by an internist. Three months later, once the spirometers had been purchased, a follow-up 2-day training led by a pulmonologist and internist was held on how to perform spirometry with a refresher course on interpreting results. Ongoing technical support was provided by an internist during the screening activities, where the team would together go over the findings of spirometry that was difficult to perform or interpret.

### Case-Finding Activities

Planning for the program began in June 2022. The first program activities began on July 28, 2022, at which time the PIH/IMB project coordinator and the NCD program manager were invited to visit the mining sites to identify the number of sites for case-finding as well as the number of participants. All mine workers from each identified site were invited to participate. The first day of site work was November 1, 2022. An average of 41 mine workers were seen each day.

#### Silicosis Education

Because silicosis is currently incurable, education about the causes and health consequences of the disease was prioritized throughout the program. The initial education activities took place at the mining sites. Teams were dispatched in 3 groups, and each team visited 1 site per day over the course of 3 days. Each team was composed of a hospital nurse, a radiology technician, 2 permanent PIH/IMB staff, and 1 WMP staff member. On-site education consisted of 1 hour of didactics, followed by an open forum for questions from mine workers. The didactic session focused on increasing awareness of silicosis, information on how the disease is acquired, protection measures, and the impact of silicosis on lung function. Among the messages emphasized was the role of routine monitoring of symptoms, chest imaging, and lung function. The radiology technician informed mine workers of the risks and benefits of chest X-ray radiation exposure.

Because silicosis is currently incurable, education about the causes and health consequences of the disease was prioritized throughout the case-finding program.

A second education session was held at the start of each case-finding day at RDH. This typically lasted 15 minutes and was led by the NCD program manager. The aim was to reinforce the causes and consequences of silicosis, as well as the importance of protective measures and routine disease surveillance. It also provided a forum for participants to ask questions about the case-finding process and have any concerns addressed without their employer present. During the registration process, participants were informed that the program was voluntary and ensured that the results would be confidential. Informed written consent was obtained at this time.

#### Chest X-Ray

Standard posterior-anterior chest X-rays were performed at the hospital to identify radiographic patterns consistent with silicosis. Images were loaded into the existing picture archiving and communication system (PACS). All X-rays were performed by a radiology technician who is a permanent staff member of RDH. The internist physician at RDH performed a preliminary review of the image and indicated whether it was normal or abnormal. For formal review, all chest X-rays were reviewed by both a Rwandan radiologist and a U.S. radiologist from Yale New Haven Hospital. Radiologists had previously been trained in the ILO’s International Classification of Radiographs of Pneumoconiosis and were instructed to classify findings based on these guidelines.^19^ A profusion score of 1/1 was used as the cutoff for chronic simple silicosis. In instances where the 2 primary radiologists disagreed on whether an X-ray was concerning for silicosis, an additional radiologist from Yale New Haven Hospital was used as an arbitrator. In cases of disagreement between the first 2 radiologists, the arbitrating third radiologist made the final decision about whether the image was concerning for silicosis. Both U.S.-based radiologists were attending-level faculty with specialization in thoracic radiology, and the Rwandan radiologist was head of a medical imaging department at a Rwandan teaching hospital. X-rays were digitized and shared with the U.S.-based radiologists for review using REDCap, while the Rwandan radiologist came to the hospital after the program was completed to perform his formal review of the images on-site through the PACS.

#### Symptom Questionnaire

A symptom questionnaire was developed to investigate common silicosis and/or TB symptoms, exposures and mine employment history, risk factors, and self-reported past medical history (Supplement). It also included questions exploring issues of chronic pain and nutrition status among mine workers. It was translated from English to Kinyarwanda by the PIH/IMB research team and then reviewed by the Rwandan clinical team to ensure that the questions were clear and comprehensible. The questionnaire was then loaded into REDCap on 6 tablets. Data collectors read all questions to the mine workers and filled in the pre-defined response choices on the form.

#### Spirometry

Six Spirobank II Basic spirometers (Medical International Research; New Berlin, Wisconsin) were purchased for this project. While spirometry has not been validated as a screening tool for silicosis, it was included to categorize patterns of lung function among miners, particularly those with radiographic silicosis, and to guide the initiation of inhaled medications. All tests were performed by the aforementioned trained nurses. In accordance with American Thoracic Society/European Respiratory Society (ATS/ERS) criteria, we targeted achieving 3 acceptable trials but would stop performing the test if either the mine worker expressed a desire to stop or if 8 attempts had been made.^20^ After the case-finding activities had ended, spirometry results were reviewed by a board-certified pulmonologist at Brigham and Women’s Hospital. ATS/ERS criteria for acceptability and repeatability were used to determine whether spirometry results were interpretable; however, the reviewing pulmonologist had discretion to determine that spirometry was clinically useful even if it did not meet ATS/ERS criteria.

#### Hypertension and Diabetes

All mine workers were tested for hypertension based on a 1-time reading using an automated blood pressure machine. A cutoff of 140/90 mmHg was used for hypertension based on the most recent Rwandan MOH national guidelines on the diagnosis and management of NCDs.^21^ Because only 1-time readings were performed, a diagnosis of hypertension could not be confirmed. Mine workers with blood pressure above the cutoff were recommended to return for a follow-up appointment for confirmatory testing and possible treatment initiation. Similarly, all mine workers had a random glucose level checked using a Codefree point-of-care glucometer and test strips. A cutoff of a random glucose greater than 200 mg/dl was used per national guidelines.^21^ We also recorded time from the last meal and self-reported prior diagnoses of hypertension and diabetes. Linkage to care and confirmatory testing for mine workers with hypertensive urgency and diabetic emergency, as well as for positive screening, are described later.

All mine workers were screened for hypertension and diabetes.

#### TB

Numerous studies have identified high rates of concurrent TB infections among individuals diagnosed with silicosis, with the prevalence of active pulmonary TB ranging from 5 to 20% in endemic countries.^22^^–^^24^ Silicosis increases the risk of developing TB by 3 to 4 times, and there is a dose-dependent relationship between quantity of silica exposure and risk of TB.^22^^,^^25^ All mine workers were surveyed with a symptom questionnaire assessing common symptoms of TB, including fevers, cough, night sweats, and weight loss, as well as for known exposures to TB. Mine workers who were determined to have an abnormal chest X-ray by on-site interpretation from an internist physician at RDH were brought to the on-site laboratory on the same day to get an Xpert MTB/RIF (Cepheid; California, USA) test performed if they were able to produce sputum. Attempts at inducing sputum were not made, but participants were requested to return the next day early in the morning to give sputum. Linkage to care is described later.

#### Program Day Flow

**FIGURE 1:**
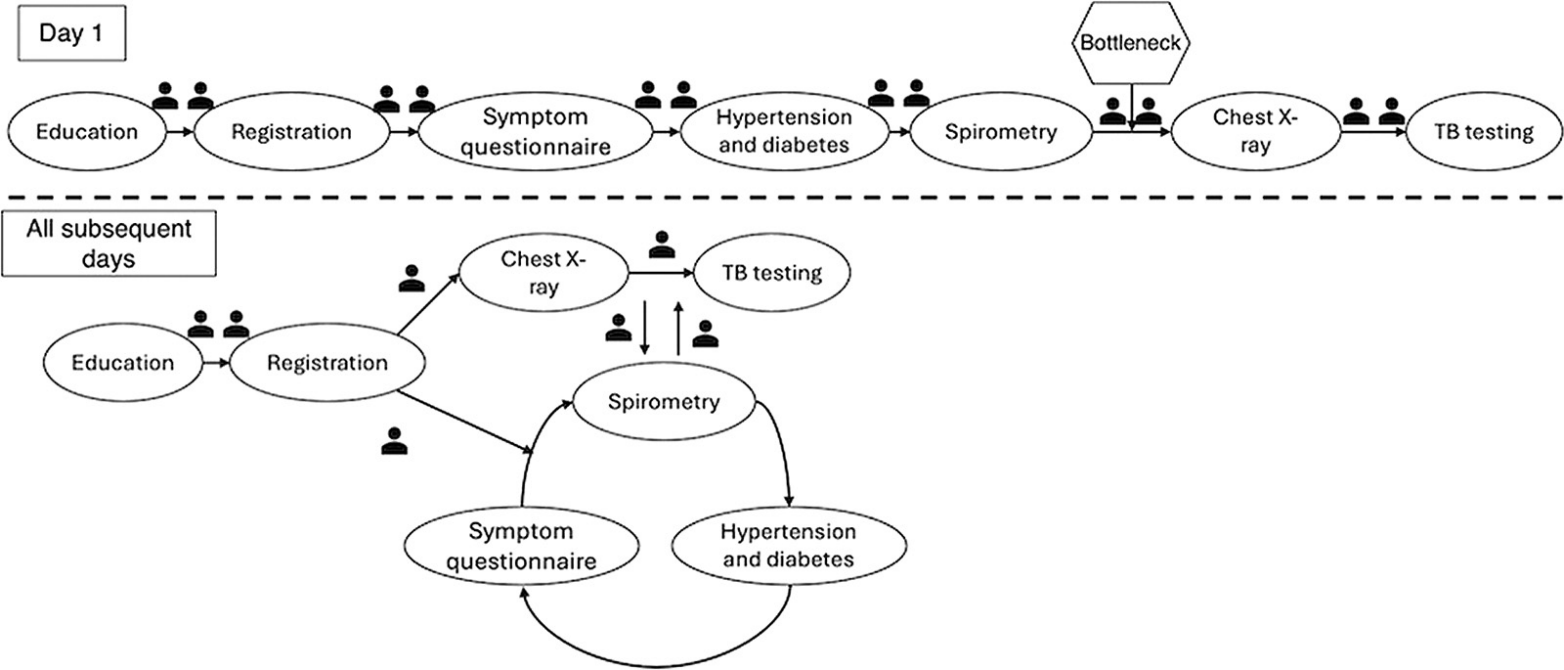
Flow of Silicosis Case-Finding Program Activities, Rwanda

On the first day of case-finding activities, all participants went through each station together consecutively leading to significant congestion in the process. Starting on day 2, half the participants were sent to the X-ray station, while the other half were equally divided between the symptom questionnaire, hypertension screening, diabetes screening, and spirometry stations. This significantly decreased the time on each day (Figure 1).

Each participant was handed a printed form with checkboxes for each station that were filled out by the station administrator to ensure completion. Additionally, the administrator indicated on the form the key findings from that station (i.e., whether exposure and symptoms of silicosis were present, whether the automatic interpretation of spirometry was abnormal, whether the mine worker had screened positive or not for hypertension or diabetes). At the end of all stations, the participant presented their completed form to the internist physician, who reviewed their X-ray at that time. If he determined it to be abnormal and the mine worker was found to have symptoms, the physician indicated on the form that Xpert MTB/RIF testing should be performed, and the mine worker would be sent directly to the laboratory. If the results were positive, they were called immediately and mandated to return to the hospital for further treatment as per the national TB case-finding and treatment protocol. All identified positive mine workers were linked to care.

### Linkage to Care

**FIGURE 2:**
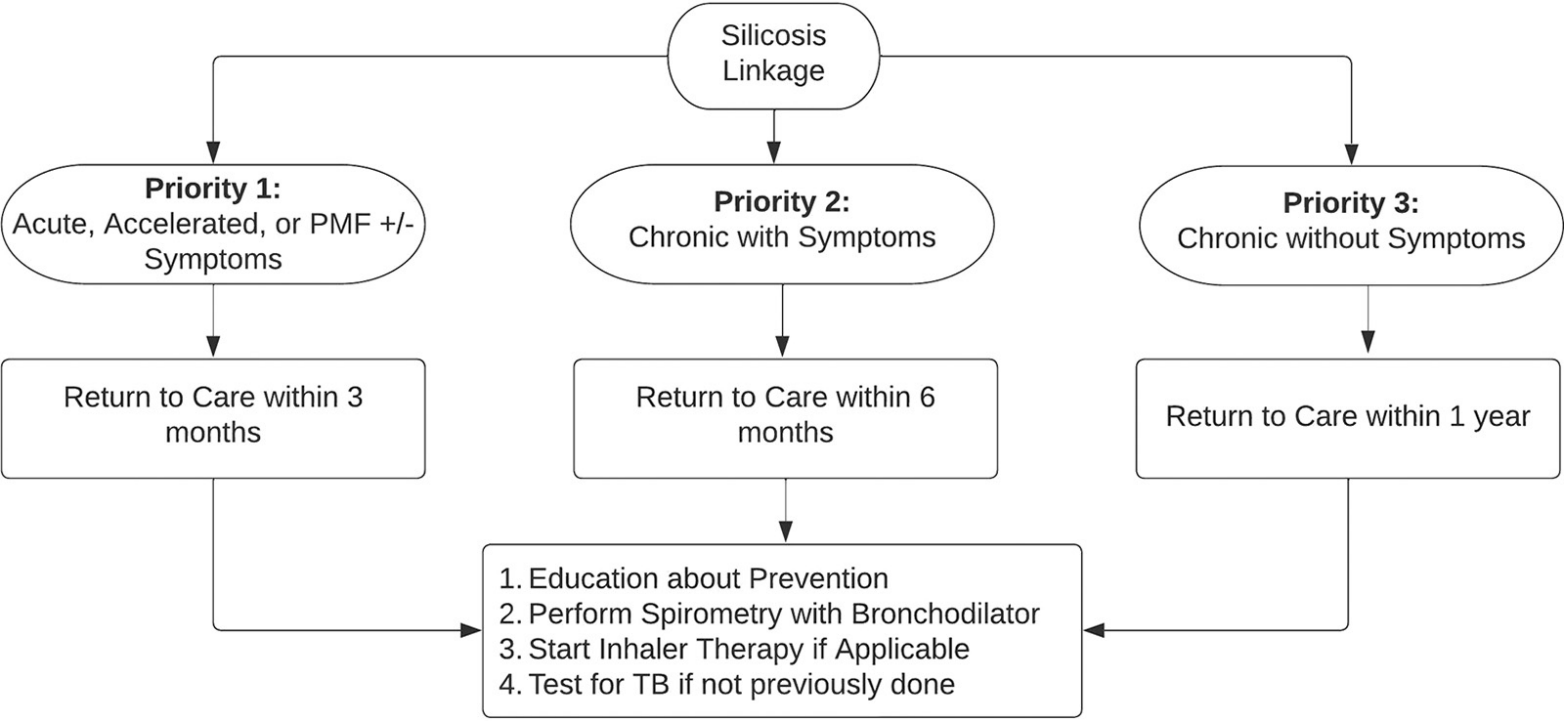
Linkage to Care Protocol for Individuals With Suspected Silicosis Abbreviation: PMF, progressive massive fibrosis.

To our knowledge, no guidelines exist for follow-up and linkage to care for individuals with suspected silicosis in resource-limited settings. In Figure 2, we detail our ongoing linkage efforts. We stratified mine workers with suspected silicosis into 3 risk groups to prioritize the timing of follow-up: (1) the highest-priority group consists of those with radiographic findings of advanced forms of silicosis (either acute, accelerated, or progressive massive fibrosis), (2) those with symptomatic radiographically chronic silicosis, and (3) those with asymptomatic radiographically chronic silicosis. All mine workers were brought back to the NCD clinic within 6 months of the completion of data analysis for disclosure of diagnosis and to arrange return visits. We intended to have return visits with the high-priority group within 3 months of completed data review, the second within 6 months, and the third group within 1 year. At the time of the return visit for all groups, the mine workers will be educated about the importance of prevention and workplace safety. Education will focus on smoking cessation, stopping mining if possible, and, if not, transitioning to lower-risk mining activities (e.g., working above ground, avoiding drilling and blasting, wearing masks), as well as the importance of routine medical care. Repeat spirometry will be performed with a bronchodilator challenge, and mine workers will be started on inhaler therapy if they have obstructive lung disease or asthma-type symptomology with normal spirometry per clinician discretion. If TB testing had not been performed previously, either due to a gap in testing or the inability to produce sputum, an attempt will be made to complete the test.

Mine workers with suspected silicosis were stratified based on screening results and linked to follow-up and care accordingly.

**FIGURE 3:**
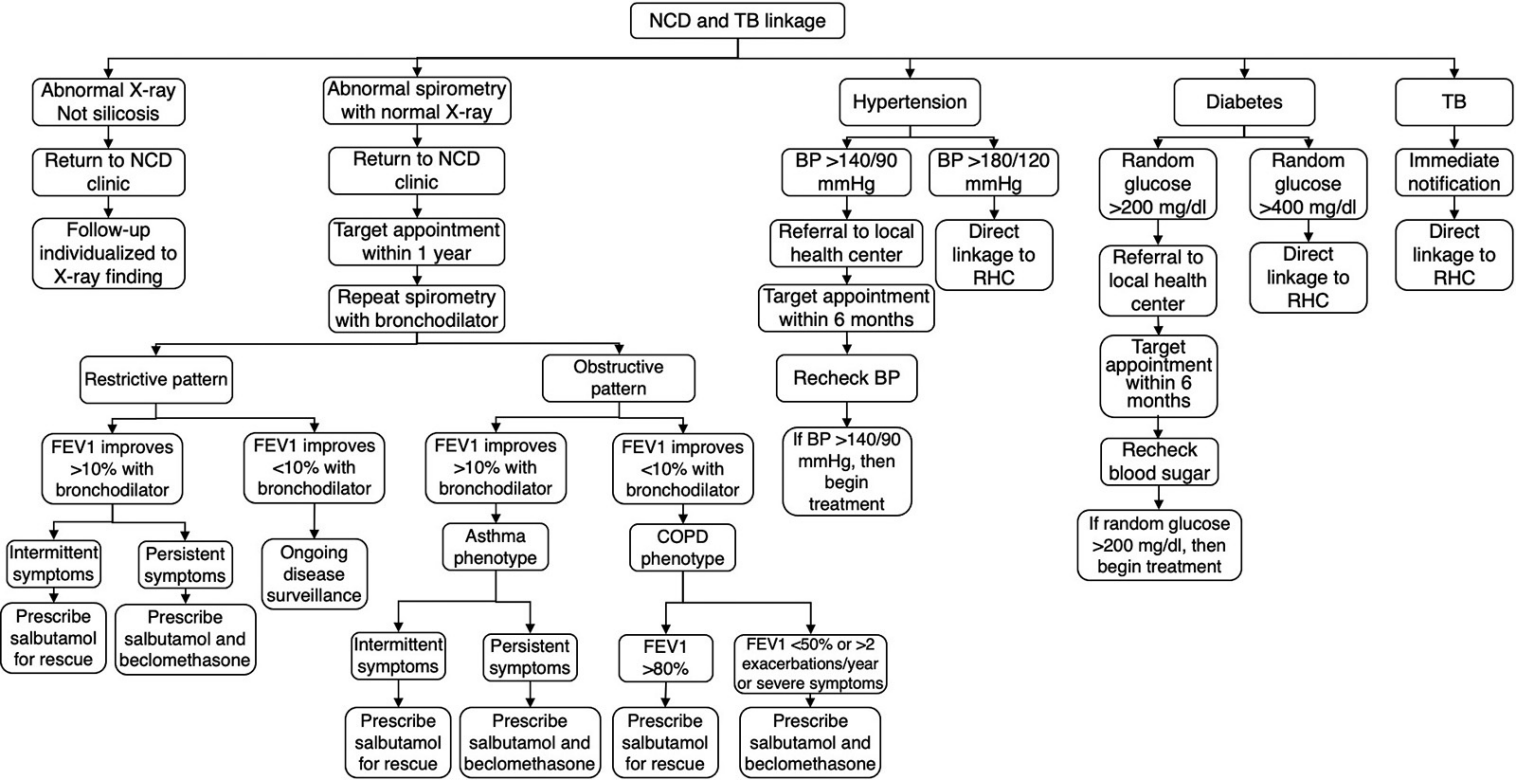
Linkage to Care Protocol for Individuals With Non-Silicosis Identified Illnesses Abbreviations: BP, blood pressure; COPD, chronic obstructive pulmonary disease; FEV1, forced expiratory volume in 1 second; NCD, noncommunicable disease clinic; RHC, Rwinkwavu Health Center.

The workflow for linkage to care for non-silicotic diseases is detailed in Figure 3. Mine workers with a positive Xpert MTB/RIF were called immediately to notify them of the result, and they were seen at the Rwinkwavu Health Center for initiation of guideline-directed therapy for TB. In Rwanda, hypertension and diabetes are primarily managed by nurses at the community health center level. Mine workers who screened positive for either disease and were not already engaged in care will be referred to their local health center for confirmatory testing and initiation of care within 6 months. Mine workers with a blood glucose greater than 400 mg/dl or a blood pressure greater than 180/120 mmHg were seen immediately for repeat testing and linkage to care. Because spirometry is known to be more sensitive than chest X-ray for many non-silicotic lung diseases, particularly obstructive lung disease, mine workers with abnormal spirometry and normal chest X-rays will be scheduled for a return visit at the NCD clinic, at which time, they will undergo repeat spirometry testing with a bronchodilator challenge and will be initiated on appropriate inhaler therapies, if indicated. While a bronchodilator challenge is typically reserved for mine workers with suspected obstructive lung disease, we plan to perform this on mine workers with either obstructive or restrictive physiology due to the known limitation of spirometry in distinguishing true restriction from pseudo-restriction (obstruction with air trapping).^26^ Additionally, follow-up will be arranged on an individualized basis for mine workers with abnormal chest X-rays that were not consistent with silicosis.

## METHODS

This descriptive analysis, which covers the period from June 2022 to February 2023, is based on debriefing interviews with key staff and stakeholders, document review of program protocols and activity reports, descriptive analysis of health management information system data captured during screening, and a retrospective review of implementation costs.

To estimate the cost of our program, we applied an ingredient-based cost analysis. We categorized costs into staff and administration, training supplies, transportation costs, screening day costs, and medical supplies and materials.^27^ Staff costs were further broken down into PIH/IMB permanent staff (nurses and program administrators), hospital clinical staff (radiology technicians, nurses, and internist physician), data collectors, and a consulting radiologist who were hired for this program. Volunteer staff time was not included in the costing calculations. For permanent staff, costs were calculated by dividing each annual salary by 260 expected working days annually and then multiplying by the number of staff members in each role as well as the number of days dedicated to the program (including training and preparation activities). An exchange rate of 1050 Rwandan francs (RWF) to 1 U.S. dollar (US$) was used. Hospital clinical staff who are employed by the MOH were paid a per diem bonus for assistance with the program that was used for calculations. Transportation costs were included for PIH/IMB staff to get to the mining sites for educational activities and meetings. However, mine workers walked a short distance from the mines to the hospital; thus, no costs were incurred. For all data collection and medical supplies/materials, costs were estimated by multiplying the unit cost by the quantity, then dividing by the expected lifespan of the item as well as 260 expected working days annually. This was then multiplied by the days used for case-finding activities. However, the chest X-ray machine and Xpert MTB/RIF machine were owned by the RDH, and PIH/IMB was charged a standard rate for each use. We use this rate multiplied by the number of mine workers tested with each modality rather than estimating cost based on the purchase and use of a new machine. We report the total cost of the program and the cost per person who participated. All analysis was performed in Microsoft Excel and Stata (version 12).

### Ethical Approval

The silicosis case-finding program was approved by the Rwandan National Ethics Committee (RNEC) 110/RNEC/2022 and by the MOH through its implementing agency, the Rwanda Biomedical Center. PIH/IMB also conducted an internal scientific and feasibility review of the study.

## KEY FINDINGS

From November 1 to December 2, 2022, 1,032 mine workers were enrolled in the case-finding program. Case-finding activities took place over 25 days, with an average of 41 people tested per day. Symptom questionnaires and demographic information were recorded for 1,025 (99.3%) participants. The median age was 32 years (interquartile range [IQR] 26–40), and 956 participants (93.3%) of the 1,025 who completed the symptom questionnaire were male. In total, 1,030 (99.8%) participants received a chest X-ray, and of these, 1,011 (98.5%) were determined to be good quality by the reviewing radiologists based on ILO criteria, with the remaining 10 being acceptable quality. Spirometry was performed on 964 (93.4%) participants, of which 765 (79.4%) received an automatically generated quality grade of A or B from the spirometer. There was marked improvement in the quality of spirometry after the first 2 days of screening, with 57% percent of tests receiving an A or B grade initially versus 82% after the first 2 days. A total of 1,021 (98.9%) mine workers were screened for hypertension and 1,017 (98.5%) were screened for diabetes. Of the 181 X-rays initially determined to be abnormal by our on-site internist physician review, 125 (69.1%) mine workers were able to produce the sputum needed for same-day Xpert MTB/RIF testing.

Our case-finding activities identified 95 mine workers with radiographic evidence of silicosis, corresponding to a prevalence of 9.2% in our sample. Five of the 125 (4%) mine workers with abnormal chest radiographs who were able to produce sputum tested positive for active TB. There were 221 (21.6%) mine workers who screened positive for hypertension, and only 12 (1.1%) had elevated glucose readings. There were 328 (32.6%) miners who identified as current or former smokers. The median duration of mine work was 7 years (IQR 3–13).

Of the 1,032 mine workers who enrolled in the case-finding program, 95 had radiographic evidence of silicosis.

### Cost of Screening

We present our ingredient-based cost analysis in the Table. The total cost of the case-finding program was estimated to be US$38,656, with more than half of the overall costs being attributed to staffing. This corresponded to a cost of US$37.49 per person tested. While we present the estimate of program costs, it does not account for the upfront capital required to perform this screening. For example, the cost of the 6 purchased spirometers was estimated to be US$75.87 based on the aggregate daily cost use over the expected lifespan of the machines; however, purchasing them required a US$7890 investment. Lastly, we had the fortune of partnering with many clinical experts who volunteered their time to this project, which reduced the actual costs incurred. Thus, while our calculated costs may overestimate the true cost as we performed our calculations using the current purchasing price for major medical equipment and did not adjust for the expected depreciation in value over time, they may also potentially underestimate true costs due to non-costed volunteer support.

**TABLE.** Ingredient-Based Cost Analysis of Silicosis Case-Finding Program Activities, Rwanda

**Category**	**Quantity**	**DaysUsed**	**Lifespan,Years**	**Total ProgramCost, US$**	**Total Cost Per PersonTested, US$**
Screening staff					
PIH/IMB permanent staff	6	50	N/A	17,973.59	17.42
Hospital clinical staff	7	30	N/A	2,631.77	2.55
Radiologist consultant	1	12	N/A	2,000.00	1.94
Data collectors	2	30	N/A	1,508.57	1.46
Subtotal				24,113.93	23.37
Training and meeting supplies					
Conference room	1	7	N/A	533.33	0.52
Food and drinks	15	6	N/A	678.57	0.66
T-shirts	100	N/A	N/A	842.86	0.82
Subtotal				2,054.76	1.99
Transport					
Driver for on-site education	1	7	N/A	114.91	0.11
Car for the field visit	1	7	15	136.75	0.13
Fuel, L	4	7	N/A	41.65	0.04
Subtotal				293.32	0.28
Data collection materials					
Tablets	6	25	3	58.97	0.06
Printing costs	82	25	N/A	195.24	0.19
Subtotal				306.59	0.30
Screening day refreshments					
Drinks	136	25	N/A	2,104.76	2.04
Snacks	140	25	N/A	500.00	0.48
Subtotal				2,604.76	2.52
Medical supplies and materials					
Glucometers	2	25	5	0.69	0.01
Strips and lancets	41	25	N/A	241.12	0.23
Blood pressure machine	2	25	3	2.98	0.00
Chest X-ray tests	41	25	N/A	6,567.81	6.36
Alcohol swabs	41	25	N/A	7.81	0.01
Cotton rolls (500 mg)	2	25	N/A	66.67	0.06
Gloves	41	25	N/A	19.52	0.02
Waste bags	10	25	N/A	142.86	0.14
Dust bins	5	25	5	3.53	0.01
Safety boxes	2	25	N/A	133.33	0.13
Spirobank II Basic	6	25	10	75.87	0.07
Disposable mouthpieces	41	25	N/A	2,050.00	1.99
Subtotal				9,312.19	9.02
Totals				38,685.55	37.49

Abbreviations: N/A, not applicable; PIH/IMB, Partners In Health/Inshuti Mu Buzima.

## DISCUSSION AND LESSONS LEARNED

Over a 25-day campaign, we successfully tested 1,032 mine workers for silicosis, hypertension, and diabetes, of which 1,014 (98.3%) completed the necessary chest X-ray and symptom questionnaire required to assess for silicosis. While all miners from the identified sites were invited to participate, we do not know the exact percentage that chose to attend (though we approximate around 90% participation), representing a potential source of selection bias, especially because participation was not renumerated. We estimated the total cost of the program to be US$38,656 and the cost per person to be US$37.49. These costs reflect our entire program, which integrated routine NCD testing, and we suspect an isolated silicosis case-finding project would have been less expensive than our estimates. While testing for other NCDs is certainly not imperative alongside occupational lung disease, our experience reflects that it can be easily integrated and likely lowers the cost of designing separate case-finding programs. However, we stress the importance of including TB case-finding in occupational lung disease surveillance, given the overlap of clinical symptoms and radiographic findings and the high rates of co-occurrence. To our knowledge, there is no source of comparison regarding the cost of case-finding programs for silicosis or other pneumoconiosis in LMICs.

We encountered several challenges during the implementation of this project, some of which we were able to solve through dynamic collaboration between stakeholders and others that serve as lessons learned for future occupational lung disease screening in similar settings.

Spirometry was introduced as a diagnostic tool at RDH for the purposes of this program. On the first day of the program, we identified high rates of spirometry results that were not of interpretable quality. The implementing team immediately contacted the U.S.-based clinical team to review these results, and together, we agreed on additional quality standards for future testing, including aiming for an A or B quality grade automatically generated by the spirometer, coaching tips to improve participant effort, targeting an expiratory time of at least 6 seconds, and continuing to test until 3 acceptable trials were completed. We then instigated routine check-ins between the U.S.-based clinical team and the implementing team to provide ongoing feedback on the quality of spirometry. Despite increased compliance with these quality benchmarks and improvement in the automatic spirometry grades after the first 2 days of the program, our ongoing formal clinical review using full ATS/ERS criteria identified many tests still did not meet full acceptability standards. In the future, when spirometry is newly introduced, we recommend a phased rollout before beginning the program to ensure that testing meets the highest quality standards. Additionally, a bronchodilator challenge was not performed for mine workers with abnormal spirometry at the time of initial testing, requiring us to repeat spirometry on these mine workers at a later date. Future efforts should anticipate integrating this into workflows. Lastly, we found that, per person, spirometry took approximately 10 to 15 minutes, making this the slowest screening activity performed, which would increase further if bronchodilator challenges were performed.

Another major challenge was the secure transfer of clinical data, particularly X-rays and spirometry results. Although the Rwandan radiologist consultant was able to come to the site to review images in the PACS, we were unable to transfer images between the Rwinkwavu and Yale New Haven hospitals’ PACS. This problem was quickly identified, and the study coordinator was able to upload screenshots of the X-rays into REDCap for review by the U.S. radiologists. This generated concerns about whether the images would be of high enough quality for accurate interpretation, so a set of sample images was sent early in the program activities for review. As reported, this system was successful, and 99.0% of images were deemed ILO grade 1 quality. Similarly, the spirometry results were uploaded as PDFs into REDCap; however, we identified that the test reports only contain a selection of the total trials attempted. We are still identifying the optimal approach to securely transfer all data collected to remote reviewers.

Integrating NCD testing for hypertension, diabetes, and non-silicosis respiratory disease into this program allowed us to successfully identify hundreds of cases of disease within this vulnerable population. It also significantly increased the amount of linkage to care that is required, providing a potential strain on the NCD infrastructure in the district. Traditionally, in Rwanda, hypertension and diabetes are first diagnosed and managed by nurses at the community health center level. We are working on a system for referring and integrating more than 200 mine workers back into this health center network of care, but these numbers exceeded our expectations given the relatively young age of the population tested. Additionally, the introduction of spirometry poses a unique challenge for linkage to care. To our knowledge, the NCD clinic in Rwinkwavu is now the only site outside of the capital, Kigali, where spirometry is available. This means that mine workers who screened positive for obstructive or restrictive lung disease will require ongoing monitoring at the NCD clinic for repeat spirometry, including with a bronchodilator challenge. Historically, asthma and COPD, which were diagnosed by history alone, were first managed at the health center level. New systems will need to be introduced to integrate the management of chronic respiratory disease between the NCD clinic at the hospital and the community health center level to not overburden either group.

Integrating NCD testing for hypertension, diabetes, and non-silicosis respiratory disease into this program allowed us to successfully identify hundreds of cases of disease within this vulnerable population.

Our case-finding program highlights the importance of ongoing stakeholder partnership, in our case, particularly between PIH/IMB, WMP, and the MOH. WMP’s identification of the need for case-finding, their support in designing and promoting a program that would be acceptable to their employees, and their assistance in creating the time during the workday for miners to come to the hospital for testing allowed for the successful implementation of this project. Similarly, the ongoing partnership between PIH/IMB and the MOH to share clinical staff and equipment allowed not only for the necessary diagnostic testing to be performed but also for us to integrate identified individuals into the existing health system. We found that investing in these partnerships was essential not only for implementation and linkage to care but also laid the groundwork for the next phase of this work, where we will continue to partner to improve safety conditions and promote the prevention of silicosis.

### Limitations

A significant limitation of this project was the missed opportunity to test all mine workers for TB instead of just those with abnormal X-rays and respiratory symptoms. This is of particular importance in such a high-risk population, where rates of TB infection are high and where silicosis serves as a risk factor for the reactivation of latent TB. Future programs should anticipate testing all mine workers able to produce sputum as well as consider testing for latent TB in this population. An additional limitation includes the use of a non-validated questionnaire for occupational exposures and respiratory symptoms; however, we are unaware of such a questionnaire existing in Kinyarwanda. Lastly, while we were able to estimate the total number of mine workers at the included mining sites based on conversations with WMP, there remains a lack of certainty around the denominator of the sample population, which, by extension, generates uncertainty regarding the response rate and adds potential selection bias to the study.

## CONCLUSION

In this article, we detail the implementation of the first-ever silicosis case-finding program in Rwanda, demonstrating the practical and economic feasibility of occupational lung disease surveillance in resource-limited settings when leveraging private-public-nongovernmental organization partnerships. We also highlight the opportunity to integrate other NCD case-finding efforts into such programs to provide more comprehensive testing and linkage to care for vulnerable populations. The results of our program emphasize the importance of improving efforts at primary and secondary prevention of silicosis among the large population of mine workers in Africa and reflect the need for partnership between the private sector, the MOH, and nongovernmental organizations to increase occupational safety standards. Our hope in publishing this article is to inspire similar surveillance activities among mining communities in LMICs.

## Supplementary Material

GHSP-D-23-00290-supplement.pdf

## Notes

**Peer Reviewed**

**First published online:** March 14, 2024.
