# Supplementary material for: Implementation and Cost Analysis of a Novel Silicosis Case-Finding Program For Mine Workers in Rural Rwanda
Source: Glob Health Sci Pract. 2024 Apr 29;12(2):e2300290. doi: 10.9745/GHSP-D-23-00290 (PMC11057793; doi:10.9745/GHSP-D-23-00290)
Supplement: GHSP-D-23-00290-supplement.pdf [file GHSP-D-23-00290-supplement.pdf]

Supplement to: Tumusime R, Miller M, Niyigena A, et al. Implementation and cost analysis of a novel silicosis case-finding program for mineworkers in rural Rwanda. Glob Health Sci Pract. 2024;12(2):e2300290. https://doi.org/10.9745/GHSP-D-23-00290

Personal Information

Amatariki yo gupima<br>Screening Date	________________________
Izina wabatijwe<br>First name	________________________
Izina wahawe n'ababyeyi<br>Last name	________________________
District/Akarere utuyemo	◯ Kayonza<br>◯ Other
If other district, specify	________________________<br>(Izina ry'akarere)
Site/Umurenge utuyemo	◯ Gahini<br>◯ Kabare<br>◯ Kabarondo<br>◯ Mukarange<br>◯ Murama<br>◯ Murundi<br>◯ Mwiri<br>◯ Ndego<br>◯ Nyamirama<br>◯ Rukara<br>◯ Ruramira<br>◯ Rwinkwavu
Cell/Akagari	________________________
Village/Umudugudu	________________________
Numero ya telephone wabonekaho ni iyihe ? Telephone number	________________________
Ese muri iyi minsi uri gukora ubucukuzi bw'amabuye y'agaciro cyangwa warabihagaritse?<br>Are you currently employed in mining?	◯ Yes<br>◯ No
Kuki waretse akazi ko gucukura amabuye y'agaciro?<br>Why are you no longer employed in mining?	◯ Respiratory Problems<br>◯ Injury<br>◯ Found another job<br>◯ Other

Izina ry'ikigonderabuzima usanzwe wivurizaho?<br><br>Name of the closest health care facility to where you live	◯ Rwinkwavu HC<br>◯ Kageyo HC<br>◯ Karama HC<br>◯ Nyamirama HC<br>◯ Ruramira HC<br>◯ Kabarondo HC<br>◯ Cyarubare HC<br>◯ Ndego HC<br>◯ Rutare HC<br>◯ Other
Niba yivuriza kukindi kigonderabuzima, andika izina ryacyo. / If other, please specify	______________________________

Supplement to: Tumusime R, Miller M, Niyigena A, et al. Implementation and cost analysis of a novel silicosis case finding program for mineworkers in rural Rwanda. *Glob Health Sci Pract*. 2024;12(2):e2300290. https://doi.org/10.9745/GHSP-D-23-00290

Rwanda : Silicosis symptoms, Spiro & X-ray

Study ID ______________________

For how long have you been working in mining? (in years) ______________________

Today 's date ______________________

SILICOSIS SYMPTOMS in the past 2 weeks)

	Yego/Yes	Oya/No
Persistent cough	◯	◯
Shortness of breath	◯	◯
Weakness	◯	◯

Do you usually bring up phlegm/Sputum when you cough?
- ◯ Sputum
- ◯ Dry cough
- ◯ No cough

When does coughing normally happen?
- ◯ When I am at work
- ◯ After climbing a mountain
- ◯ When I am walking so fast
- ◯ When walking in normal way
- ◯ Anytime, even at rest
- ◯ None
- ◯ Other

If other, please specify ______________________

When does shortness of breath normally happen?
- ◯ When I am at work
- ◯ After climbing a mountain
- ◯ When I am walking so fast
- ◯ When walking in normal way
- ◯ Anytime, even at rest
- ◯ None
- ◯ Other

If other, please specify ______________________

Have you ever worked in the construction industry?
- ◯ Yes
- ◯ No

For how long have you worked in construction ? ______________________ (In months)

Do you live with someone who smokes?
- ◯ Yes
- ◯ No

Have you ever smoked?
- ◯ Yes
- ◯ No

Question	Response
Are you currently a smoker?	◯ Yes<br>◯ No
For how long have you been tobacco-free?	______________<br>(in months)
How long have you been smoking?	______________<br>(in months)

Spirometry results

Question	Response
Spirometry quality grade	◯ A<br>◯ B<br>◯ C<br>◯ D<br>◯ E<br>◯ F
Forced Vital Capacity (FVC)	______________
Forced expiratory volume in 1 second (FEV1)	______________
FEV1/FVC ratio	______________
Peak flow (liter/minute)	______________
Peak flow (percent)	______________
Interpretation of spirometry results?	◯ Normal<br>◯ Restrictive abnormality<br>◯ Obstructive abnormality
If restrictive or obstructive, show the severity	◯ Mild<br>◯ Moderate<br>◯ Severe

Manual interpretation of Spirometry by Boston Team

Question	Response
Manual Spirometry Results interpretation	◯ Normal<br>◯ Abnormal<br>◯ Uninterpretable
If abnormal, is it restrictive or obstructive?	☐ Restrictive<br>☐ Obstructive

If restrictive, show the severity	◯ Mild restrictive<br>◯ Moderate restrictive<br>◯ Severe restrictive
If obstructive, show the severity	◯ Mild obstructive<br>◯ Moderate obstructive<br>◯ Severe obstructive

Local interpretation of Chest X-ray

Was the X-ray done?	◯ Yes<br>◯ No
Tentative interpretation of X-ray results by Rwinkwavu internist/GP	◯ Normal<br>◯ Abnormal
Record the severity of chest-x-ray abnormalities	◯ Mild<br>◯ Moderate<br>◯ Severe
Clinical recommendation	◯ Immediate follow up<br>◯ Increase protective measures<br>◯ GeneXpert test for TB
If GeneXpert test was done, what is the results of the TB test?	◯ Positive<br>◯ Negative
Name of a local health provider who interpreted the x-ray	______________________

Chest X-Ray evaluation

By Radiologist

First name of the radiologist who interpreted the X-ray images	______________________
Technical quality of CXR image	◯ Good /Grade 1<br>◯ Acceptable with no defect /Grade 2<br>◯ Acceptable with some defect /Grade 3<br>◯ Unacceptable /Grade 4
Is Silicosis suspected?	◯ Yes<br>◯ No
If silicosis is suspected, show the severity of silicosis	☐ Acute<br>☐ Chronic<br>☐ Progressive massive fibrosis<br>☐ Accelerated

Size of opacity observed	◯ small (= < 1cm)<br>◯ large (>1 cm)<br>◯ Both large and small opacity
Profusion scores of small opacity	◯ 0/0<br>◯ 0/1<br>◯ 1/0<br>◯ 1/1<br>◯ 1/2<br>◯ 2/1<br>◯ 2/2<br>◯ 2/3<br>◯ 3/2<br>◯ 3/3<br>◯ 3/+
Shape of small opacity	◯ regular<br>◯ irregular
Predominant size of regular small Opacity	◯ p - diameter of [< =1.5mm)<br>◯ q - diameter of [1.5 - 3mm]<br>◯ r - diameter of [3 -10mm]
Secondary shape and size of the observed small Opacity	◯ p ◯ q ◯ r ◯ s<br>◯ t ◯ u
Record the size , if irregular small opacity	◯ s - width of [< =1.5mm]<br>◯ t - width of [1.5 - 3mm]<br>◯ u - width of [3 -10mm]
if CXR has large opacities, please record the category of the large opacity	◯ Cat A (< 50mm)<br>◯ Cat B (>50mm, but < right upper zone)<br>◯ Cat C (> = right upper zone)
Is patient suspected to have any other respiratory illnesses?	◯ Yes<br>◯ No
Which other respiratory illness is suspected?	◯ Pneumonia<br>◯ Tuberculosis<br>◯ lymphadenopathy<br>◯ Fibrosis of lung<br>◯ Emphysema<br>◯ Lung collapse<br>◯ Bronchiaectasis<br>◯ Pleural effusion<br>◯ lung cancer<br>◯ Other
If other illness, specify	__________________________________

Supplement to: Tumusime R, Miller M, Niyigena A, et al. Implementation and cost analysis of a novel silicosis case finding program for mineworkers in rural Rwanda. *Glob Health Sci Pract*. 2024;12(2):e2300290. https://doi.org/10.9745/GHSP-D-23-00290

TB screening

Date ______________________

TB CLINICAL SCREENING

	Yego/Yes	Oya/No
Cough lasting more than 2 weeks and with sputum	○	○
Fever and night sweats in the past 2 weeks	○	○
Unexplained weight loss in the past 2 weeks	○	○
Chest pain in the past 2 weeks	○	○
Loss of appetite in the past 2 weeks	○	○
Ever diagnosed, or met or lived with someone with TB	○	○

Supplement to: Tumusime R, Miller M, Niyigena A, et al. Implementation and cost analysis of a novel silicosis case finding program for mineworkers in rural Rwanda. *Glob Health Sci Pract*. 2024;12(2):e2300290. https://doi.org/10.9745/GHSP-D-23-00290

Silicosis Research Data

Ukorera kuyihe site?<br><br>Working Site	◯ Gahengeli A<br>◯ Gahengeli B<br>◯ Gahengeli C<br>◯ Gahengeli D<br>◯ Rutonde A<br>◯ Rutonde B<br>◯ Raveri<br>◯ Gihinga<br>◯ Nyarunazi A<br>◯ Nyarunazi E<br>◯ Nyarunazi D<br>◯ Migera A<br>◯ Migera B<br>◯ Gahushyi A<br>◯ Gahushyi B<br>◯ Gahushyi C<br>◯ Gahushyi D<br>◯ Gahushyi E<br>◯ Cross-site<br>◯ Other/Ahandi
Have you worked from any other mining site?	______________________
Ufite imyaka ingahe y'amavuko?<br><br>How are old you?	______________________<br>(Date of Birth)
Sex/ igitsina	◯ Male/ Gabo<br>◯ Female/ Gore
Irangamimerere ryawe ni irihe?<br><br>Marital Status	◯ Single/Ingaragu<br>◯ Married/Narashatse<br>◯ Cohabitating/Bitanyuze mumategeko<br>◯ Divorced /Twaratanye<br>◯ Widow/Narapfakaye
Ese wize amashuri angahe?<br><br>What is your highest level of Education?	◯ No school<br>◯ Attended but did not finish primary school<br>◯ Completed primary school
Ubana n'abantu bangahe mu rugo?<br><br>Family Size	______________________
Niwowe mukuru w'umuryango?<br><br>Are you the head of the Household?	◯ Yes<br>◯ No

Uba mukihe kiciro cy'ubudehe?

Ubudehe category

- ◯ Category 1
- ◯ Category 2
- ◯ Category 3
- ◯ Category 4
- ◯ I do not know

Waba warigeze ubwirwa na muganga ko urwaye uburwayi bukurikira? Subiza yego cyangwa Oya/ Have you ever been told by a doctor or nurse that you have the following disease?

	Oya/No	Yego/Yes
Igituntu / TB	◯	◯
Umusonga	◯	◯
COVID-19	◯	◯
Asima /Asthma	◯	◯
Diyabete / Diabetes	◯	◯
Ubwandu bwa SIDA / HIV	◯	◯
Umuvuduko w'amaraso/ Hypertension	◯	◯
Uburwayi bw'umutima / Heart Failure	◯	◯
Canceri / Cancer	◯	◯
Wigeze ubagwa / Prior Surgery	◯	◯
Silikoze / Silicosis	◯	◯

Part three: Working environment and risk factors of silicosis

Ni kuyihe myanya cyangwa imirimo ikorwa mukazi ko gucukura waba warigeze ukoraho? [Hitamo ibisubizo byose bishoboka]

Which stations have you ever worked in? Select all that apply

- ◯ Gutobora urutare/ Drilling
- ◯ Gucukura/ Excavation
- ◯ Guturitsa Intambi/ Blasting
- ◯ Kumena itaka/ Soil disposal
- ◯ Kuronga umusaruro/ Harvest washing

Wamaze igihe kingana iki utobora amabuye?(Imyaka)

Length of time of working as a driller

____

Wamaze igihe kingana iki ukora umurimo wo gucukura (gucukura umwobo wo mu kirombe)?

____

Length of time working as an excavator

Wamaze igihe kingana iki ukora umurimo wo guturitsa intambi?

Length of time working in blasting ______________________

Wamaze igihe kingana iki ukora umurimo wo kumena itaka? ______________________

Length of time working in soil disposal

Wamaze igihe kingana iki ukora umurimo wo kuronga umusaruro? ______________________

Length of time working in harvest washing?

3.4 Ibibazo bikurikira birabaza niba ufite ibikoresho by'ubwirinzi mu kazi ukora ko gucukura amabuye y'agaciro. / These questions ask about protective equipment to use at work in mining.

	Oya	Yego
Ese waba ujya uhabwa uturinda ntoki? / Do you have gloves to wear at work?	○	○
Waba ujya uhabwa udupfukamuwa twabugenewe mugucukura amabuye yagaciro/ Do you have face masks for mining to wear at work?	○	○
Waba ufite igisarubeti cy'akazi? / Do you have overalls to wear at work?	○	○
Iyo urangije akazi utaha ucyambaye igisarubeti? / Do you go home wearing the same clothes you worked in?	○	○
Waba ufite inkweto z'akazi Bote zabugenewe) / Do you have boots to wear at work?	○	○
Waba ufite ingofero y'ubwirinzi? / Do you have a Helmet to wear at work?	○	○

Niba uhabwa uturindantoki, wavugako mukwezi gushize wadukoresheje kangahe?

If you have gloves at work, how often do you wear them?

- ○ Nta na rimwe (Never)
- ○ Gake cyane (Rarely)
- ○ Rimwe na rimwe (Sometimes)
- ○ Hafi ya buri gihe (Often times)
- ○ Buri gihe (All the times)

Niba uhabwa agapfukamunwa kabugenewe, wavugako mukwezi gushize wagakoresheje kangahe?

If you have face masks at work, how often do you wear them?

- ○ Nta narimwe (Never)
- ○ Gake cyane (Rarely)
- ○ Rimwe na rimwe (Sometimes)
- ○ Hafi ya buri gihe (Often times)
- ○ Buri gihe (All the times)

Niba uhabwa ingofero y'ubwirinzi, wavugako mukwezi gushize wayambaye igihe cyose uri mukazi kangahe?<br><br>If you have overalls at work, how often do you wear them?	◯ Nta na rimwe (Never)<br>◯ Gake cyane (Rarely)<br>◯ Rimwe na rimwe (Sometimes)<br>◯ Hafi ya buri gihe (Often times)<br>◯ Buri gihe (All the times)
Mukwezi gushize, ni kangahe wakarabye ivumbi rikuriho mbere y'uko utaha mu rugo?<br><br>If you have change your clothes at work before coming home, how often do you do so?	◯ Nta na rimwe (Never)<br>◯ Gake cyane (Rarely)<br>◯ Rimwe na rimwe (Sometimes)<br>◯ Hafi ya buri gihe (Often times)<br>◯ Buri gihe (All the times)
Iyo wakoze, ni amasaha angahe umara mukazi ?<br>How many hours per day do you spend at work?	______________________
Ese mu kirombe ukoramo, iyo mutobora urutare mukoresha amazi?<br>In the mines, do you use water drilling techniques?	◯ Yes<br>◯ No<br>◯ I don't know
Amazi mukoresha mutobora/mumena amabuye aba ahari ku kihe gipimo?<br>How frequently do you use water drilling techniques?	◯ Gake cyane (Rarely)<br>◯ Rimwe na rimwe (Sometimes)<br>◯ Hafi ya buri gihe (Often times)<br>◯ Buri gihe (All the times)
Kuva watangira gucukura amabuye y'agaciro, hashize igihe kingana iki mutangiye gukoresha amazi muri gutobora amabuye?<br>How long have you been using water when drilling stones/rocks?	______________________

Mu byumweru 2 bishize, waba waragize ububabare budasanzwe mu bice by'umubiri bikurikira?
In the past two weeks have you experienced pain in the following body parts?

	Yego/Yes	Oya/No
Umugongo wo hasi / Lower back	◯	◯
Umugongo w'ahasubira hejuru Amatako/ Hips or Thighs	◯	◯
Ibitugu / Shoulders	◯	◯
Ijosi/ Neck	◯	◯
Agatuza/ chest	◯	◯

Ubujana/ Wrists or hands	○	○
Ibirenge/Feet	○	○
Amavi / knees	○	○

Ukoresheje igipimo cyo kuva kuri 1-10, aho rimwe ari ububabare buke 10 bukaba ububabare bukabije, garagaza ikigero cy'ububabare wagize mu bice by'umubiri wavuze haruguru? / On a scale of 1 to 10 with 10 being the worst pain you have ever felt, how severe is the pain?

Ububabare bw'umugongo wo hasi wagize wabuha amanota angahe ku icumi?

Lower back pain scores/ 10

____

Ububabare bw'umugongo wo hasi ubumaranye igihe kingana iki?

For how long have you had lower back pain?

____
(Show if it's years months or days)

Ububabare bw'umugongo wahagana hejuru wabuha amanota angahe ku icumi?

upper back pain scores/ 10

____

Ububabare bw'umugongo wahagana hejuru ubumaranye igihe kingana iki?

For how long have you had upper back pain ?

____
(Show if it's years, months or days)

Ububabare bwo mumaguru /amatako wagize wabuha amanota angahe ku icumi?

Thigh/leg pain scores/ 10

____

Ububabare bwo mumaguru /amatako ubumaranye igihe kingana iki?

For how long have you had thigh/leg pain ?

____
(Show if it's years, months or days)

Ububabare bwo mu bitugu wagize wabuha amanota angahe ku icumi?

Shoulder pain scores/ 10

____

Ububabare bwo mu bitugu ubumaranye igihe kingana iki?

For how long have you had Shoulder pain ?

____
(Show if it's years, months or days)

Ububabare bw'ijosi wagize wabuha amanota angahe ku icumi?

Neck pain scores/ 10

____

Ububabare bw'ijosi ubumaranye igihe kingana iki?<br><br>For how long have you had neck pain ?	______________________<br>(Show if it's years, months or days)
Ububabare bwo mugatuza wagize wabuha amanota angahe ku icumi?<br><br>Chest pain scores/ 10	______________________
Ububabare bwo mugatuza ubumaranye igihe kingana iki?<br><br>For how long have you had chest pain ?	______________________<br>(Show if it's years, months or days)
Ububabare bwo mu bujana wagize wabuha amanota angahe ku icumi?<br><br>Wrist pain scores/ 10	______________________
Ububabare bwo mu bujana ubumaranye igihe kingana iki?<br><br>For how long have you had wrist pain?	______________________<br>(Show if it's years, months or days)
Ububabare bwo mu mavi wagize wabuha amanota angahe ku icumi?<br><br>Knee pain scores/ 10	______________________
Ububabare bwo mu mavi ubumaranye igihe kingana iki?<br><br>For how long have you had knee pain ?	______________________<br>(show if it's years, months or days)
Utekereza ko ubu bubabare bwatewe n'iki ?<br><br>What is the suspected cause of most pain reported above?	◯ Mining related<br>◯ Related to prior non-mining job<br>◯ Disease-related<br>◯ Unknown
Waba warigeze ugira imvune/uvunika kuva watangira y'agaciro?<br><br>Have you ever sustained an injury since you started working in mining?	◯ Yes, a work-related injury amabuye<br>◯ Yes, a non-work related injury<br>◯ No

Supplement to: Tumusime R, Miller M, Niyigena A, et al. Implementation and cost analysis of a novel silicosis case finding program for mineworkers in rural Rwanda. *Glob Health Sci Pract*. 2024;12(2):e2300290. https://doi.org/10.9745/GHSP-D-23-00290

Ese aho ukorera waba ugira ingaruka zo kunyeganyega kw'ingingo z'umubiri bitewe no gukoresha imashini cyangwa gukorera ahantu hari imashini zihinda cyane?<br><br>Have you been exposed to vibration at work?	◯ Yes<br>◯ No
Niba ugirwaho ingaruka no gutitira kw'imashini z'aho mukorera, wavugako imashini zititira ku gihe kigero?<br><br>How would you rate the level of vibration?	◯ Ikigero gito cyane / minimal<br>◯ Ikigero kiri mu rugero / moderate<br>◯ Ndifashe / neutral<br>◯ Ikigero kiri hejuru/ Severe<br>◯ Ikigero gikabije cyane/ very severe
Ni ibihe bice by'umubiri wawe bigirwaho ingaruka no gutitira bitewe no guhinda kw'imashini z'aho mukorera?<br><br>What parts of your body were affected by vibration?	◯ Gutitira umubiri wose igihe ari jye uri gukoresha imashini / whole body<br>◯ Gutitira kw'ukuboko gusa kuri gukoresha imashini /hands and arms
